# Combined V-Y Fasciocutaneous Advancement and Gluteus Maximus Muscle Rotational Flaps for Treating Sacral Sores

**DOI:** 10.1155/2016/8714713

**Published:** 2016-06-06

**Authors:** Hyun Ho Han, Eun Jeong Choi, Suk Ho Moon, Yoon Jae Lee, Deuk Young Oh

**Affiliations:** Department of Plastic and Reconstructive Surgery, College of Medicine, The Catholic University of Korea, Seoul 06591, Republic of Korea

## Abstract

The sacral area is the most common site of pressure sore in bed-ridden patients. Though many treatment methods have been proposed, a musculocutaneous flap using the gluteus muscles or a fasciocutaneous flap is the most popular surgical option. Here, we propose a new method that combines the benefits of these 2 methods: combined V-Y fasciocutaneous advancement and gluteus maximus muscle rotational flaps. A retrospective review was performed for 13 patients who underwent this new procedure from March 2011 to December 2013. Patients' age, sex, accompanying diseases, follow-up duration, surgical details, complications, and recurrence were documented. Computed tomography was performed postoperatively at 2 to 4 weeks and again at 4 to 6 months to identify the thickness and volume of the rotational muscle portion. After surgery, all patients healed within 1 month; 3 patients experienced minor complications. The average follow-up period was 13.6 months, during which time 1 patient had a recurrence (recurrence rate, 7.7%). Average thickness of the rotated muscle was 9.43 mm at 2 to 4 weeks postoperatively and 9.22 mm at 4 to 6 months postoperatively (*p* = 0.087). Muscle thickness had not decreased, and muscle volume was relatively maintained. This modified method is relatively simple and easy for reconstructing sacral sores, provides sufficient padding, and has little muscle donor-site morbidity.

## 1. Introduction

Pressure sores are challenging to medical and nursing staff because they are slow to heal, prone to recurrence, and difficult to reconstruct [1–3]. The sacral region is one of the most common locations for such wounds, and surgical methods are required when conservative measures fail [4].

**Figure 1:**
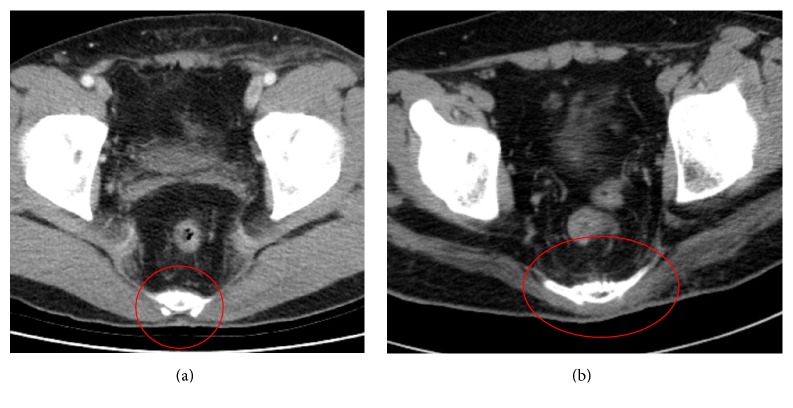
(a) Computed tomographic (CT) image of the sacrococcygeal joint of a healthy ambulatory individual. Gluteus muscle volume is well maintained, but the area around the sacrum appears thinned. (b) CT image of a patient who received modified gluteal fasciocutaneous flaps using a rotational muscle flap. The muscle appears well padded.

Gluteal flaps have been used for treating sacral sores since 1970 [5]. Since then, diverse treatment methods have been developed; the most popular methods are a musculocutaneous flap and a fasciocutaneous flap [6–8]. A musculocutaneous flap has the advantage of sufficient padding at the pressure point, but, on the other hand, it has limitation on arc of rotation, and, over time, the padded muscle portion may experience atrophic degeneration. In addition, for ambulatory patients, donor-site morbidity could be a potential problem [7]. In particular, the central portion of the gluteus muscle is relatively thinner than the lateral side normally, so padding of the sacral region with a standard musculocutaneous flap is not easy (Figure 1).

A fasciocutaneous flap is relatively thinner than a musculocutaneous flap but has advantages during flap rotation and inset. Furthermore, compared with a musculocutaneous flap, a fasciocutaneous flap has lower rate of recurrence [7, 9, 10].

Here, we propose a new method for treating sacral sores that combines the advantages of fasciocutaneous and musculocutaneous flaps: combined V-Y fasciocutaneous advancement and gluteus maximus muscle rotational flaps. The advantage of this method is that this procedure is simple and that sufficient padding on the defect area is possible, as the fasciocutaneous flap and the relatively thick central portion of the gluteus muscle flap are combined for coverage with plentiful thickness, reducing the recurrence risk. There is less limitation of the rotation arc due to separate coverage of the fasciocutaneous flap, which makes it easier to inset the flaps. The disadvantage of this method is probability of slightly longer operation time than a classic fasciocutaneous flap. We present clinical cases in which our technique was successfully utilized to treat sacral sores.

## 2. Materials and Methods

Our Institutional Review Board approved this study (KC15RISI0133). Between March 2011 and December 2013, a total of 13 patients were admitted to our institution with a sacral sore and were treated with a modified gluteal fasciocutaneous flap. All patients were graded with stage IV pressure sore with extension to the bone.

Data regarding each patient's age, sex, reason for being bedridden, and follow-up duration were collected. Surgical details, including defect size, operating time, and estimated blood loss, were recorded. Complications, including reoperation, dehiscence, flap necrosis (partial or total), wound infection, sinus formation, and donor-site morbidity, also were recorded. If a sore developed again within 1 year at the same surgical site, we considered it a recurrence [6].

Computed tomography (CT) was performed postoperatively at 2 to 4 weeks and again at 4 to 6 months to measure the thickness of the padded muscle at the sacrococcygeal area and to assess whether muscle thickness was preserved. CT images were recorded at a slice spacing of 1 mm. The thickness of the muscle was measured on the basis of the parameter, sacrococcygeal joint, and the value was compared. The Wilcoxon signed-rank test was used to test the null hypothesis that mean pre- and postoperative muscle thicknesses were equal. A *p* value of <0.05 was considered statistically significant. Statistical analyses were performed using the Statistical Package for the Social Sciences version 13.0 (SPSS, Inc., Chicago, IL, USA).

## 3. Surgical Method

**Figure 2:**
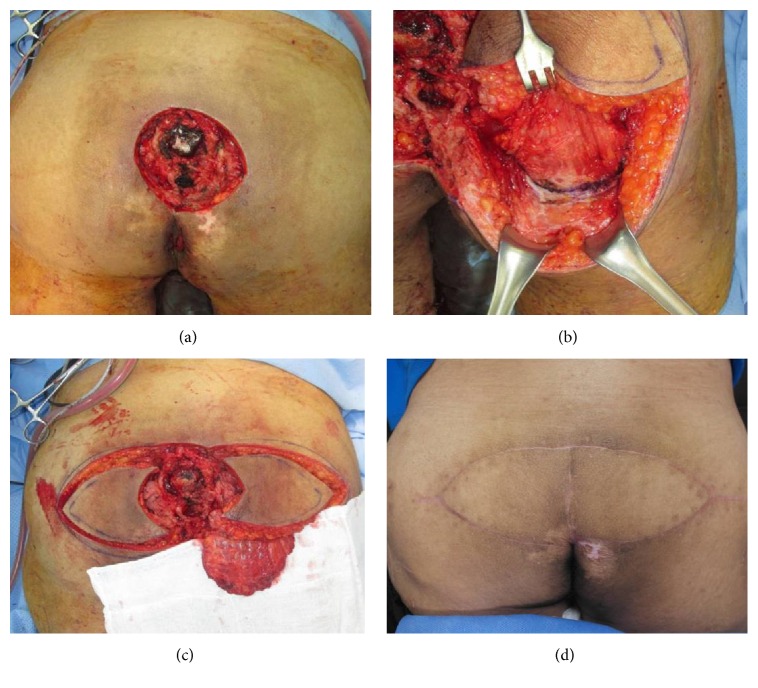
(a)-(b) The defect site and muscle flap design after elevating the fasciocutaneous flap. From the inferior margin of the fasciocutaneous flap to the inferior region of the buttock, 10–15 cm was dissected to sufficiently reveal the inferior portion of the gluteus maximus. (c) Then, a U-shaped muscle flap that was 80% to 90% of the defect size was elevated. (d) Clinical photograph at 5 months postoperatively.

**Figure 3:**
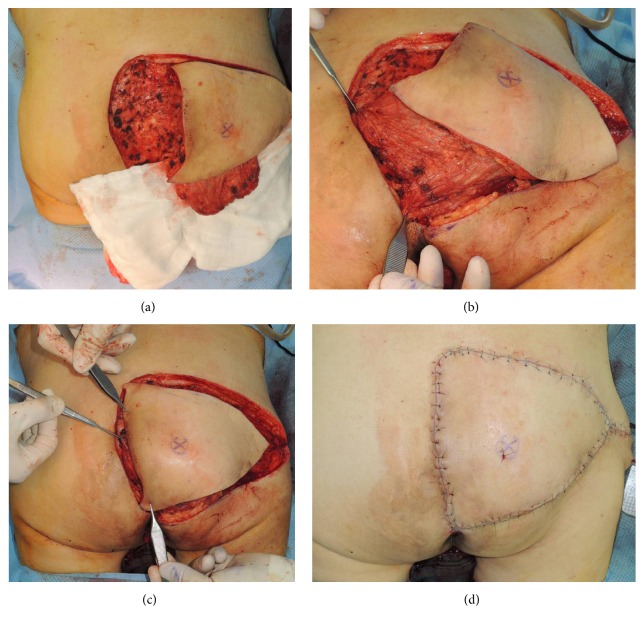
(a) The muscle flap immediately after elevation. (b) After the muscle was rotated to the defect site, the deep fascia on the opposite side was sutured. Then, the fasciocutaneous flap was rotated likewise. (c) The rotated fasciocutaneous flap was moved to the defect site, advanced, and sutured. It was not kinked with the muscle flap and was inset without trouble. (d) After closure.

**Figure 4:**
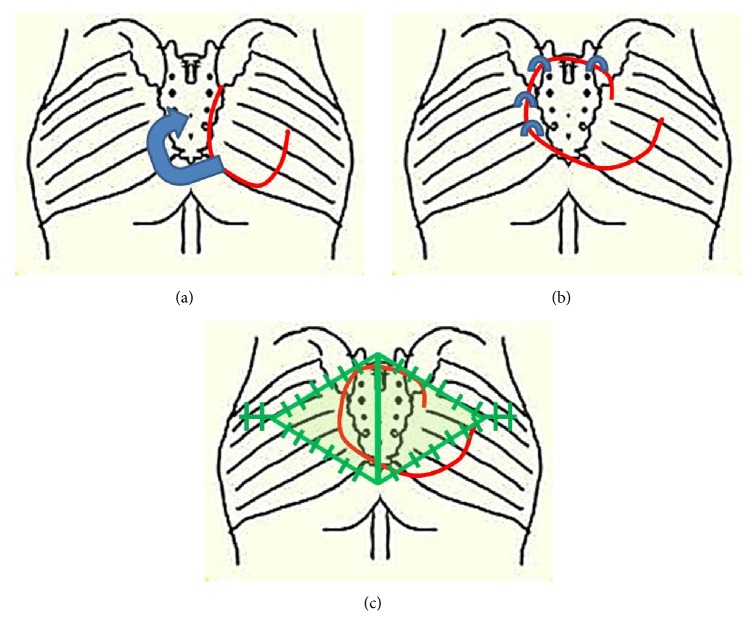
Surgical procedure diagram of muscle division and rotation to the defect. (a) The muscle was mobilized starting from distal portion into a U-shaped flap leaving approximately 5 to 6 cm attached proximally. (b) After rotation, the mobilized muscle flap was sutured to the muscle fascia on the opposite side or remnant around anchoring. (c) Then, the previously elevated fasciocutaneous flap was slid to the medial side and inset in a V-Y advancement manner.

Operations were performed with the patient in a prone position under general anaesthesia. Absolute debridement was performed with elimination of devitalized tissue. Based on the sacral bone condition (whether osteomyelitis was observed, which was present in most patients), the necrotic unviable bone tissue was removed until healthy medullary bleeding was seen at the base of the debrided wound. Tissue culture was confirmed in all operations. In our experience, after debridement, a fasciocutaneous flap elevated on one side was sufficient to close defects of up to 12 to 15 cm in diameter. For larger defects, or in cases in which closure of the unilateral flap could only be achieved under tension, bilateral flaps were elevated. Then, from the inferior margin of the fasciocutaneous flap to the inferior portion of the buttock, a 10 to 15 cm further dissection was performed in order to adequately reveal the inferior portion of the gluteus maximus muscle. Afterward, a U-shaped muscle flap that was 80% to 90% of the defect size was elevated (Figures 2 and 3). Because the inferior gluteal artery is exposed during the procedure, dissection must be performed with caution so as not to damage the artery. Approximately 30~40% of the entire gluteus muscle was harvested from the medial and lower portion, preserving 100% of the lateral insertion portion and part of the upper portion. The muscle was mobilized starting from distal portion into a U-shaped flap leaving approximately 5 to 6 cm attached proximally (Figure 4(a)). After rotation, the mobilized gluteus maximus muscle flap was sutured to the muscle fascia on the opposite side or remnant around anchoring with Vicryl #2-0 without direct fixation to the bone (Figure 4(b)). Then, the previously elevated fasciocutaneous flap was slid to the medial side and inset in a V-Y advancement manner (Figure 4(c)). All muscle flaps were elevated from only one side, although the bilateral V-Y fasciocutaneous flap covering the wide external surface was used for defected areas of more than 150 cm^2^. All operations were performed by a single surgeon.

## 4. Postoperative Management

All patients were put on a standard postoperative regimen that included a closed suction drain maintained for at least 1 week and a low residue diet in the first week. Antibiotics were used in accordance with the results of tissue culture from deep tissue or resected bone debris after debridement performed during operation. Antibiotic therapy containing intravenous and oral medication was continued for about 4 weeks. The position was changed every 2 hours strictly. Ambulatory patients began walking at 2 weeks and sitting at 4 weeks for 1 hour intervals once a day, then gradually increasing the length and frequency of sitting periods by about 2 hours.

## 5. Results

**Figure 5:**
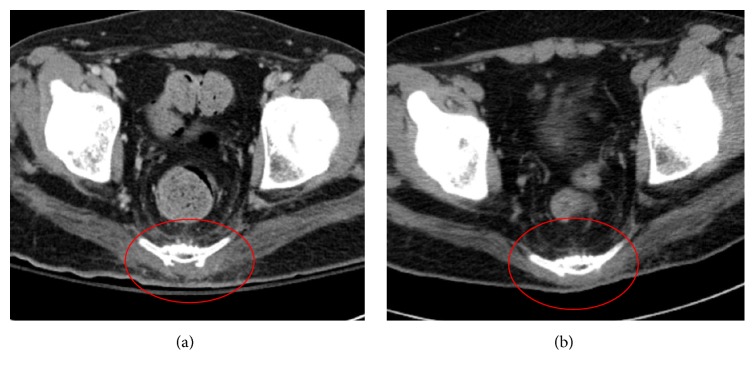
(a) Computed tomographic (CT) image at 3 weeks postoperatively. (b) CT image at 5 months postoperatively, showing maintenance of muscle depth with no change.

Demographic data, including sex, age, comorbidities, follow-up duration, defect size, operating time, estimated blood loss, complications, recurrence, and muscle width, are displayed in Table 1. Minor complications, including seroma and hematoma, occurred in 3 patients, but all patients were completely healed within 1 month with conservative care. One of the patients experienced hematoma at the elevated area of the inferior muscle flap, which subsided with conservative care. With the exception of 1 patient who died 9 months after surgery, all patients were followed for more than 12 months, with an average follow-up period of 13.6 months. During follow-up, 1 patient experienced a recurrence, resulting in a 7.7% recurrence rate. Average thickness of the rotated muscle was 9.43 mm at 2 to 4 weeks postoperative, and 9.22 mm at 4 to 6 months postoperative (*p* = 0.087). We were able to confirm that even at 4 to 6 months after surgery, the width of the muscle was not greatly reduced and the volume was relatively maintained (Figure 5).

## 6. Discussion

Various methods for pressure sores have been developed [11–13]. One of them, a gluteus maximus myocutaneous flap, has been favoured by most surgeons for reconstructing sacral pressure sores [5, 7, 14]. Due to its sufficient padding, there have been several reports that it can reduce recurrence [6, 9]; however, it has such disadvantages as limited flap mobility, loss of muscle, and increased blood loss. Sameem et al. [15] reported that there was no difference in recurrence rates between a gluteus maximus myocutaneous flap and a fasciocutaneous flap, while Koshima et al. [16, 17] reported that the transferred muscle portion of the flap showed remarkable atrophic change over the long term.

If we consider the anatomy of the sacral area, the muscle becomes thinner closer to the centre, and, in the centre, no muscle is present and the subcutaneous tissue becomes thinner. If we do not consider these characteristics and use a fasciocutaneous flap or a myocutaneous flap with classic V-Y advancement, the central area that requires the most padding is padded by thin tissue. As a result, the coverage becomes weak, leading to a higher chance of recurrence. Moreover, the tissue is difficult to suture. Koshima et al. [16, 17] also reported that it is common for muscle atrophy to occur.

We proposed a method that combines the advantages of a fasciocutaneous flap and a musculocutaneous flap. The muscles utilized are not the ones used in the existing classic gluteal myocutaneous flap method, and the medial and inferior portions of the gluteus maximus are split and then rotated, resulting in sufficient muscle thickness and coverage. During the operation, 30~40% of the entire gluteus muscle was harvested from the medial and lower portion conserving 100% of the lateral insertion portion and part of the upper portion without detachment. The muscle flap was elevated with the lower central portion vertical incision remaining in the upper portion with a length of 5~6 cm. The detachment of the lower fibers of the gluteus muscle can weaken limb adduction in nonparalytic patients. However the other adductor, the pectineus, and gracilis muscles can substitute. In practice, the ambulatory patients had no problems in ambulation postoperatively. It is beneficial for ambulatory patients because donor-site morbidity is small, and the dissected area is not wide; thus, the surgery itself is not too complicated.

According to our results, even at 4 to 6 months after surgery, muscle thickness was maintained, which proves that padding was sufficiently done. A fasciocutaneous flap has the advantage of having great mobility, and, especially in the recently commonly used superior gluteal artery perforator (SGAP) flap, the rotation arc has almost no limits. In this method, the muscle is split and the flap is inset; thus, similar to an SGAP flap, mobility can be adequately acquired. In addition, because only 30% to 40% of the gluteus maximus is used, in the case of recurrence, the chance of using the same muscles for surgery can be preserved.

Most patients with pressure sore are paraplegic or tetraplegic by cause of spinal cord injury [18] Such patients are more likely to be neglected from timely intervention. When that occurs, the possibility of multiple pressure sores including ischial and/or trochanteric area is increased. In addition, taking into consideration that the pressure sores are likely to occur, it is necessary to consider the possibility of 2nd and 3rd operation. Even though the gluteus muscle should be mobilized, at least half the proportion must be spared for the possibility of recurrence.

Most patients with grade IV pressure sore on sacral areas are accompanied with osteomyelitis [19–21]. Treatment protocol includes adequate debridement and antibiotics therapy for 6 weeks [22]. When reconstructed under the method as the author has described, the lesion with osteomyelitis lies in the centre of muscle flap. We speculate that rich blood supply from the overlying muscle flap might provide positive effect on fighting the infection.

In short, this method is simple and easy technically. Sufficient padding on the defect area is possible as fasciocutaneous advancement flap and thick central portion of gluteus maximus muscle flap compared to conventional coverage of relatively thin origin portion are combined for coverage of the defect area with ample thickness, especially in sacral sores going to the bone. The thickness of the muscle flap is well maintained reducing the recurrence risk of pressure sore. Muscle donor-site morbidity is tolerable causing fewer difficulties in postoperative ambulation with detachment of partial medial lower portion of muscle with minimal complications. There is less limitation of the rotation arc due to independent coverage of fasciocutaneous flap which facilitates in setting the flaps. When preparing for the possibility of 2nd and 3rd operation in patients prone to pressure sores recurrence, upper portion of the ipsilateral gluteus muscle can be conserved. The padding of the central portion of muscle, which has rich blood supply, to the debrided bone might help fight dormant infection. On the other hand, the disadvantage of this method is the possibility of longer incision if bilateral fasciocutaneous flap is used and the operation time can be prolonged. The use of fasciocutaneous flap on the contralateral side can cause difficulty in reoperation with fasciocutaneous flap or gluteus maximus myocutaneous flap from the contralateral side causing possible scarring or adhesion. The division of the lower fiber of gluteus maximus muscle can weaken limb adduction although the other adductors, the pectineus, and gracilis muscles can substitute this function.

The limited number of patients and short period in follow-up are the drawbacks of this study. In fact, practicing a regular follow-up in bed-ridden patients is not easy and measuring the thickness of gluteus muscle after 6 months postoperatively has clinical significance. Inconsistent fasciocutaneous flap coverage, unilateral or bilateral, is also a concern. Nevertheless most patients underwent the bilateral flaps with an average 164 cm^2^ defect size, which can minimize the variables needed for drawing a overall conclusion.

The modified method we proposed is relatively simple and easy for reconstructing sacral sores, provides sufficient padding, and has little muscle donor-site morbidity. Once sufficient padding is established in cases like this, a takeaway from this report would be to study the recurrence rates compared with the classic fasciocutaneous flap.

## Figures and Tables

**Table 1:** Patient data and results.

Patient	Sex/age, y	Reason for being bedridden	Paralytic state	Follow-up time, month	Defect size, cm^2^	Operating time, min	Estimated blood loss, cc	Complications	Recurrence	Muscle thickness, mm (2–4 weeks postop)	Muscle thickness, mm (4–6 months postop)
1	M/62	Spinal cord injury	Quadriplegia	12	84	90	50	None	None	8.42	8.19
2	M/72	Cerebrovascular accident	Hemiplegia	13	289	83	100	Seroma	None	7.33	7.45
3	M/26	Spinal cord injury	Paraplegia	16	120	180	50	None	None	11.2	10.42
4	M/55	Spinal cord injury	Quadriplegia	12	80	102	50	None	None	9.82	9.75
5	F/55	Spinal cord injury	Quadriplegia	15	195	90	75	None	None	10.25	10.33
6	M/60	Spinal cord injury	Quadriplegia	12	210	150	100	None	None	8.82	8.76
7	M/76	Cerebrovascular accident	Quadriplegia	14	77	115	75	None	None	9.52	9.49
8	F/43	Spinal cord injury	Paraplegia	13	156	180	100	Seroma	Once	7.22	7.88
9	F/13	Brain tumour	Quadriplegia	18	208	90	75	None	None	10.95	10.43
10	M/81	Cerebrovascular accident	Hemiplegia	9	342	180	150	None	None	9.87	9.78
11	F/64	Cerebrovascular accident	Quadriplegia	12	120	120	75	None	None	8.38	8.12
12	F/34	Anorexia nervosa	None	15	77	95	50	None	None	11.24	11.06
13	F/39	Spinal cord injury	Paraplegia	16	169	88	100	Hematoma	None	9.52	8.32

Average				13.6	164	130.25	81		7.7% (1/13)	9.43	9.22
										*p* > 0.05	

F: female; M: male; postop: postoperative.
